# The Feasibility of Antioxidants Avoiding Oxidative Damages from Reactive Oxygen Species in Cryopreservation

**DOI:** 10.3389/fchem.2021.648684

**Published:** 2021-02-26

**Authors:** Xiangjian Liu, Yiming Xu, Fenglin Liu, Yuxin Pan, Lu Miao, Qubo Zhu, Songwen Tan

**Affiliations:** ^1^ Xiangya School of Pharmaceutical Sciences, Central South University, Changsha, China; ^2^ School of International Pharmaceutical Business, China Pharmaceutical University, Nanjing, China

**Keywords:** cryopreservation, cryoprotectants, reactive oxygen species, oxidative damages, antioxidants

## Abstract

Cryopreservation prolongs the storage time of cells and plays an important role in modern biology, agriculture, plant science and medicine. During cryopreservation, cells may suffer many damages, such as osmotic dehydration, large ice puncture and oxidative damages from reactive oxygen species (ROS). Classic cryoprotectants (CPAs) are failing to dispose of ROS, while antioxidants can turn ROS into harmless materials and regulate oxidative stress. The combination of antioxidants and CPAs can improve the efficiency of cryopreservation while negative results may occur by misuse of antioxidants. This paper discussed the feasibility of antioxidants in cryopreservation.

## Introduction

Cryopreservation is a technique for preserving cells at low temperatures, which can prolong their storage time. However, organisms are easy to be damaged during freezing for the following two reasons: osmotic damage and mechanical damage. Osmotic damage is caused by the freezing of the extracellular solution, leading to increases in the concentrations of the solutes. Subsequently, the cells are damaged by osmotic dehydration. Mechanical damage refers to the puncture damage of cells by sharp ice crystals (Yang et al., 2017). Therefore, many cryoprotectants (CPAs) have been developed to reduce damages. Permeable CPAs, such as DMSO(Ock and Rho, 2011) and glycerol (Rogers et al., 2018), can enter cells to adjust osmotic pressure and reduce osmotic damage. Impermeable CPAs, such as antifreeze protein (Xiang et al., 2020) can decrease the size of extracellular ice crystals to reduce mechanical damage. The addition of CPAs can improve the efficiency of cryopreservation.

However, recent studies have shown that oxidative stress occurs in cells during cryopreservation. Oxidative stress refers to a state of imbalance between oxidation and anti-oxidation, which is caused by the massive production of reactive oxygen species (ROS) in extreme conditions such as low temperatures in cells (Evangelista-Vargas and Santiani, 2017). Cellular antioxidants, such as glutathione and thioredoxin, can resist ROS by participating the reduction process when the concentration of ROS is low (Yang et al., 2018; Alhayaza et al., 2020). However, the large amount of ROS produced during cryopreservation can cause the oxidation of proteins, lipids and nucleic acids (Chen and Li, 2020). These may cause irreversible damages to cells and even lead to apoptosis (Len et al., 2019). Classic permeable and impermeable CPAs are failing to reduce oxidative damage to cells.

Antioxidants, such as ascorbate acid (Mathew et al., 2019), glutathione (Diengdoh et al., 2019), mitoquinone (Sui et al., 2018), salidroside (Alotaibi et al., 2016), resveratrol (Longobardi et al., 2017) and so forth, can resist the oxidative stress and reduce the damages from ROS. Therefore, antioxidants and CPAs can be used together to comprehensively reduce the harm in cryopreservation. It must be noted that the misuse of antioxidants could cause negative effects. So appropriate antioxidants must be carefully selected in cryopreservation. In this paper, the source, species, properties, mechanisms and damages of ROS are introduced in detail. The results of the combination with CPAs and antioxidants are also concluded to promote the development of cryopreservation.

## Reactive Oxygen Species

### Properties

ROS mainly includes superoxide anion radical $\left({\text{O}}_{2}^{•-}\right)$, hydrogen peroxide (H_2_O_2_) and hydroxyl radical (·OH) in cryopreservation (Huang et al., 2018). Under normal physiological conditions, ROS can regulate cell growth and differentiation (Len et al., 2019). However, ROS could be overwhelmingly produced at low temperature and cause damages to cells (Jia et al., 2017). Generally, ${\text{O}}_{2}^{•-}$ derives from complex Ⅲ in mitochondria. Coenzyme Q intermediate $\cdot {\text{Q}}^{-}$ easily transfers electrons to O_2_ and ${\text{O}}_{2}^{•-}$ is formed (Finkel and Holbrook, 2000). ${\text{O}}_{2}^{•-}$ is moderately active with a short half-life (about 1 μs), and it is the main source of other ROS in cells (Sharma et al., 2012). The high solubility of ${\text{O}}_{2}^{•-}$ makes it difficult to penetrate the cell membrane (Mumbengegwi et al., 2008), and ${\text{O}}_{2}^{•-}$ cannot react with most biomolecules (Halliwell, 2006). Under the existence of superoxide dismutase (SOD) or by spontaneous dismutation, ${\text{O}}_{2}^{•-}$ can react with H^+^ to form H_2_O_2_(Marrocco et al., 2017). H_2_O_2_ is moderately active with a half-life of 1 ms. Unlike other ROS, H_2_O_2_ has no charge and can enter cells easily through aquaporin. So H_2_O_2_ can cause damage in multiple places due to its strong membrane permeability (Bienert et al., 2007). ${\text{O}}_{2}^{•-}$ and H_2_O_2_ can produce ·OH by the Haber-Weiss reaction. ·OH contains an active unpaired single electron that can react with most biological molecules. So ·OH is considered to be the most toxic ROS (Sharma et al., 2012).

### Damages

In cryopreservation, the damage caused by ROS can be attributed to lipid peroxidation (Banday et al., 2017), protein oxidation (Mostek et al., 2017) and DNA damage (Ladeira et al., 2019). Lipid peroxidation (LPO) refers to the decomposition of lipids into aldehydes such as 4-hydroxynonenal (4-HNE) and malondialdehyde (MDA) under the action of ROS. The content of MDA in cells can reflect the degree of LPO (Tsikas, 2017). LPO seriously affects cells’ function due to lipid is an important part of cell membranes (Uchendu et al., 2010). Besides, MDA is highly toxic and can react with nucleic acids and proteins, further causing damages to cells (Long et al., 2009). Proteins can be converted into carbonyl proteins by ROS, and the content of carbonyl in proteins can indicate the degree of protein oxidation (Li et al., 2010). Protein oxidation can induce DNA damage, lipid damage, cell secondary damage, and lower enzyme efficiency (Davies, 2016). Furthermore, gene mutation, double/single strand breaking occur in DNA in the presence of ROS (Len et al., 2019), causing serious damage such as apoptosis (Zhao et al., 2016). Comet assay is a standard test to quantitatively detect the degree of DNA damage (Ladeira et al., 2019). All the damages caused by ROS can seriously affect the physiological function of cells and reduce the efficiency of cryopreservation.

## The Effects of Antioxidants

Antioxidants are powerful substances to counter ROS. The use of specific antioxidants at appropriate concentrations can significantly reduce the damages from ROS and improve the efficiency of cryopreservation. However, the wrong use of antioxidants can result in negative results.

### Positive Effects

**FIGURE 1:**
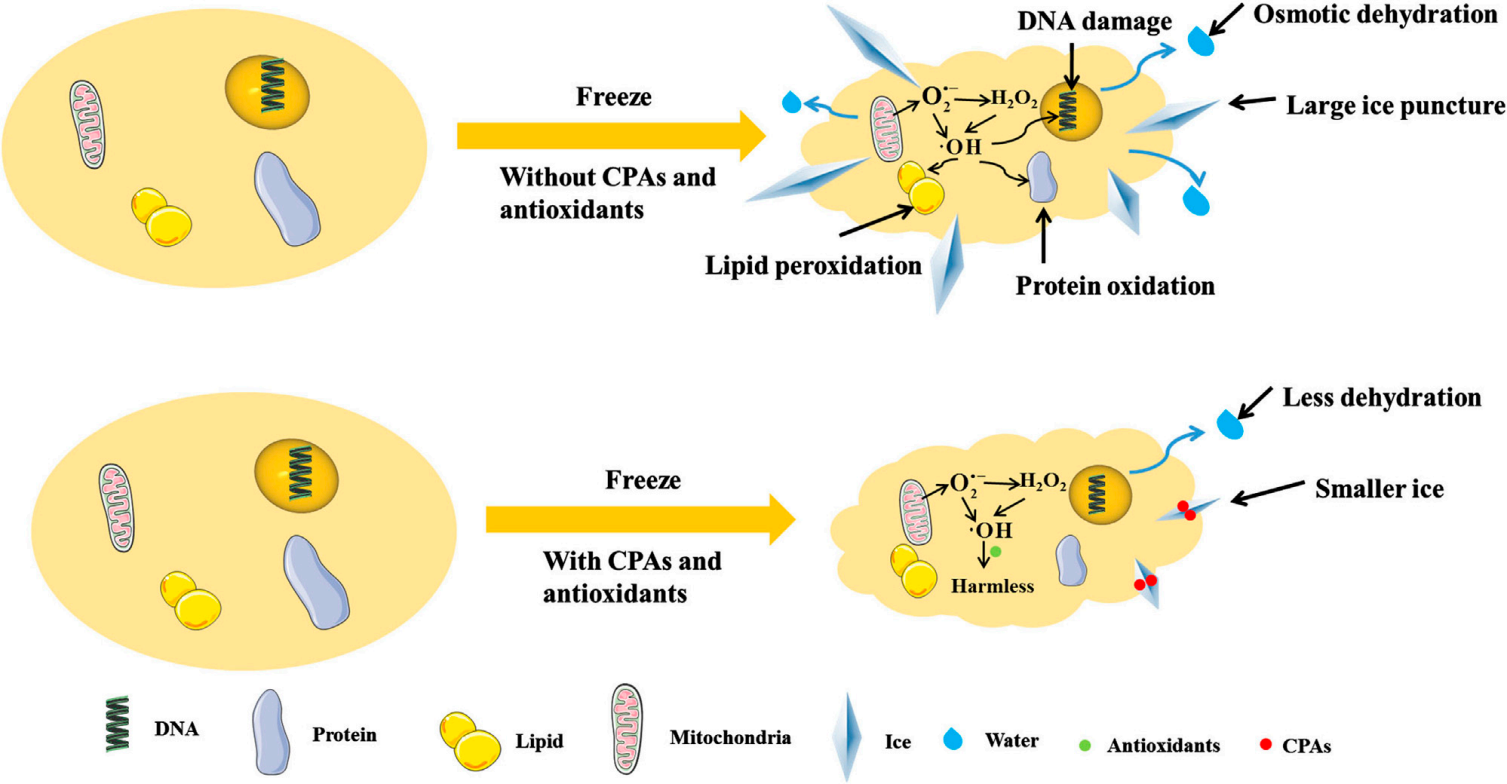
The freeze cell with/without CPAs and antioxidants. CPAs can adsorb in ice surface to inhibit ice growth, and regulate osmotic pressure to reduce dehydration. Antioxidants can reduce the production of ROS and turn ROS into harmless materials, so as to relieve the damages by ROS. The antioxidants and CPAs can use together to reduce the damages from cryopreservation comprehensively.

In cryopreservation, antioxidants can reduce oxidative stress (Mathew et al., 2019), regulate the synthesis of mitochondrial proteins (Banday et al., 2017), decrease ROS production (Zhu et al., 2019), clear intracellular ROS (Len et al., 2019), enhance the activity of antioxidant enzyme (Azadi et al., 2017), resist to LPO and DNA fragmentation (Yousefian et al., 2018). Specifically, for germ cells such as sperm, antioxidants can increase motility parameters (Toker et al., 2016), acrosomal integrity (Lone et al., 2018), mitochondrial membrane potential (Fontoura et al., 2017) and pregnancy rates (Ren et al., 2018). Therefore, the combination of antioxidants and CPAs may reduce the damages to cells caused by osmotic dehydration, large ice puncture and ROS during freezing and thawing, and improve the efficiency of cryopreservation (as shown in Table 1 and Figure 1).

**TABLE 1:** The applications of antioxidants.

Antioxidants	CPAs	Cryopreservation objects	Positive results	Cryopreservation method	References
Ascorbate acid	Sucrose and PVS2^a^	Kiwifruit shoot tips	Lipid peroxides↓	Droplet vitrification^b^	Mathew et al. (2019)
			Protein carbonyls↓		
			Regeneration↑		
	TEYCAFG^c^	Cross-bred cattle bull semen	Live spermatozoa↑	4°C for 4 h, programmatically cool to −140°C and transfer into LN	Singh et al. (2020a)
			Acrosomal integrity↑		
			Sperm abnormalities↓		
			MDA↓		
			SOD↑		
Glutathione, ascorbate acid and vitamin E	Sucrose	Mint shoot tips	Stable samples percentage↑	Vitrification	González-Benito et al. (2016)
Catalase and malate dehydrogenase	None	*Paeonia* and *Magnolia* pollen	Germination rate↑	Vitrification	Jia et al. (2018)
			SOD↑		
			ROS and MDA↓		
Glutathione	Sucrose and PVS2	Orchids protocorms	Post-thaw recovery↑	Encapsulation-vitrification	Diengdoh et al. (2019)
Single-wall carbon nanotubes	PVS2	*Agapanthus praecox* embryogenic callus	ROS↓	Vitrification	Ren et al. (2020)
			Cells oxidative injury↑		
			Survival rate↑		
N-acetyl-L-cysteine	DMSO^d^	Human cord blood nucleated cells	ROS↓	Cool at 1–3°C/min to −80°C, then transfer into LN^e^	Makashova et al. (2016)
			Viability↑		
			Preservation rate↑		
Catalase and α-tocopherol	DMSO and fetal bovine serum	Spermatogonial stem cells	ROS↓	Store at −80°C for 1 day then transfer into LN	Aliakbari et al. (2017)
			The number of cells↑		
			Cells quality↑		
			Viability↑		
Mitoquinone	VS83^f^	Heart valve tissue	Tissue viability↑	Programmatically cool to −130°C for 24 h and transfer into LN for 2 mouths	Sui et al., (2018)
Salidroside	Glycerol or trehalose	Sheep red blood cells	Hemolysis↓	Vitrification	Alotaibi et al. (2016)
			Protein oxidation↓		
			Lipid oxidation↓		
Taurine	Tris extender^g^	Crossbred ram sperm	Percent sperm motility↑	Programmatically cool to −140°Cand transfer into LN	Banday et al. (2017)
			Live sperm count↑		
			MDA↓		
			Glutathione↓		
Leptin	SpermFreeze^h^	Human sperm	DNA fragmentation↓	Store at LN vapor phase then transfer into LN	Fontoura et al. (2017)
			Antioxidant enzymes activity↑		
MitoTEMPO	SpermFreeze	Human spermatozoa	Sperm motility↑	Place in vapor LN and transfer into LN	Lu et al. (2018)
			Viability↑		
			Membrane integrity↑		
			Mitochondrial membrane potential↑		
Coenzyme Q_10_	Soybean lecithin-based extender^i^	Buck spermatozoa	Total motility↑	4°C for 2 h, LN vapor phase for 12 min; last transfer into LN	Yousefian et al. (2018)
			Sperm viability↑		
			Plasma membrane functionality↑		
			Sperm abnormality↓		
			Mitochondrial activity↑		
Lycopene	Triladyl^j^	Bovine sperm	Mitochondrial activity↑	4°C for 2 h,programmatically cool to −140°Cand transfer into LN	Tvrda et al. (2017)
			ROS↓		
			Protein carbonyl↓		
			Lipid peroxidation↓		
			DNA damage↓		
Lycopene and alpha-lipoic acid	Extender II^k^	Goat spermatozoa	Sperm motility↑	4°C for 2 h,programmatically cool to −5°Cand transfer into vapor LN	Ren et al. (2018)
			Acrosome integrity↑		
			Membrane integrity↑		
			Mitochondrial activity↑		
			Pregnancy rates↑		
α-Tocopherol and ascorbic acid	DMSO, glucose and bovine serum albumin	Spermatozoa of Atlantic salmon	Lipid peroxidation↓	Programmatically cool from 4°C to −120°C	Figueroa et al. (2018)
			Glutathione peroxidase↑		
			Catalase activity↑		
			ROS↓		
			Mitochondrial membrane potential↑		
			Percentage of motility↑		
Melatonin	BotuCrio^l^	Equine sperm	Percentage of sperm cells ↑	Programmatically cool to −140°C and transfer into LN	Lançoni et al. (2018)
			Mitochondrial membrane potential↑		
Resveratrol	Optidyl^m^	Goat semen	The total motility↑	5°C for 4 h, place in vapor LN for 10 min, last transfer into LN	Lv et al. (2019)
			Progressive motility↑		
			Membrane and acrosome integrity↑		
			Mitochondrial activity↑		
			Percentage of viable spermatozoa↑		
			ROS↓		
Aloe vera	Tris-egg-yolk-citric-acid-fructose-glycerol extender	Bull semen	Progressive motility↑	4°C for 4 h, programmatically cool to −140°Cand transfer into LN	Singh et al. (2020b)
			Live spermatozoa↑		
			Acrosomal integrity↑		
			MDA↓		

^a^ PVS2: plant vitrification solution 2:30% (w/v) glycerol, 15% (w/v) ethylene glycol and 15% (w/v) dimethyl sulphoxide.

^b^ Vitrification: a method for cryopreservation which can make the intracellular and extracellular environment form a glass-like shape, usually requiring high CPA concentration and rapid cooling (Rienzi et al., 2016).

^c^ TEYCAFG: Tris-Egg-Yolk-Citric-acid-Fructose-Glycerol extender.

^d^ DMSO: dimethyl sulfoxide.

^e^ LN: Liquid nitrogen.

^f^ VS83: vitrification solution 83%:4.65 M dimethyl sulfoxide, 4.65 M formamide, and 3.30 M 1,2-propanediol.

^g^ Tris extender (Tris citric acid buffer 73 ml; fructose 1.25 g; egg yolk 20 ml; glycerol 7 ml; penicillin G sodium 80,000 IU; streptomycin 100 mg).

^h^ SpermFreeze: a commercial CPA(Vitrolife, Sweden).

^i^ Soybean lecithin-based extender: (3.07 g Tris, 1.26 g fructose, 1.68 g citric acid in 100 ml distilled water), soybean lecithin 1.5% (w/v) and glycerol 5% (v/v).

^j^ Triladyl: a commercial CPA (Minitub GmbH, Tiefenbach, Germany).

^k^ Extender II: 6 mM glucose, 600 mM Tris, 190 mM citric acid, 0.4 g/ml streptomycin, 2000 IU/ml penicillin, egg yolk (15%, v/v) and glycerol (5%, v/v) in 200 ml deionized water.

^l^ BotuCrio: a commercial CPA (Botupharma, Botucatu, SP, Brazil)ptidyl: a commercial CPA(Biovet, France).

^m^ Optidyl: a commercial CPA(Biovet, France).

### Negative Effects

There are some negative effects of using antioxidants in cryopreservation. For instance, when ascorbic acid is used for cryopreservation of *Aranda* Broga Blue orchid, the growth regeneration percentage will be reduced from 5 to 1.7% (Khor et al., 2020). In the cryopreservation of human semen, the addition of ascorbic acid, vitamin E, and L-carnitine can adversely affect sperm motility, especially at high concentrations (Banihani and Alawneh, 2019). The reason may be that antioxidants not only reduce ROS but also have negative effects on the endogenous antifreeze mechanism of cells (Khor et al., 2020). Furthermore, the high concentrations of antioxidants transform cells from oxidative stress to reductive stress, which may also have negative effects on the structure and function of cells (Bisht and Dada, 2017). It is noticeable that the use of antioxidants in cryopreservation is not always satisfactory.

## Conclusion and Prospect

Cryopreservation is more and more widely used nowadays. Many CPAs have been developed to reduce damages during freezing and thawing. ROS produced at low temperatures can cause lipid peroxidation, protein oxidation and DNA damage, seriously affect the structure and function of cells, and even cause cell apoptosis. Traditional CPAs cannot resist ROS. Antioxidants can decrease oxidative stress, reduce the production of ROS, convert ROS into harmless substances, and increase the activity of ROS enzymes. Therefore, the use of antioxidants and CPAs in cryopreservation may increase cells’ survival rate, motility and reproductive capacity, reduce lipid peroxidation, protein oxidation and DNA damage, decrease the osmotic and mechanical damages by ice, so the efficiency of cryopreservation is increased. It must be noted that the use of antioxidants does not always have a positive effect, especially when the concentration of antioxidants is relatively high. This may be that antioxidants can destroy the natural antifreeze mechanism of cells and transform cells from oxidative stress to reductive stress. This suggests that antioxidants are a double-edged sword, and good results only occur when antioxidants are used properly.

At present, there are the following research directions of antioxidants in cryopreservation.

1. Expanding applications. Currently, antioxidants are mainly used for the cryopreservation of cells and plant tissues. In the future, antioxidants can be used cautiously in the cryopreservation of human tissues and organs to promote the development of organ transplantation, regenerative medicine and cryomedicine.
2. Exploring mechanisms. The microcosmic interaction between antioxidants and ROS in cells is still unclear. The study of mechanisms can guide the development and application of antioxidants.
3. Using untapped antioxidants. Many natural and artificial antioxidants may have potential in cryopreservation and not be used yet. Using untapped antioxidants with proper CPAs may increase the efficiency of cryopreservation cheaply and effectively.
4. Revealing effective conditions. Sometimes antioxidants may cause negative results in cryopreservation. For the development of antioxidants in cryopreservation, it is important to reveal the conditions that positive results will occur.
